# Assessment of extended-spectrum β-lactamase (*ESBLs*) – producing *Escherichia coli* from minced meat of cattle and swab samples and hygienic status of meat retailer shops in Jimma town, Southwest Ethiopia

**DOI:** 10.1186/s12879-019-4554-6

**Published:** 2019-10-28

**Authors:** Mengistu Abayneh, Getnet Tesfaw, Kifle Woldemichael, Moti Yohannis, Alemseged Abdissa

**Affiliations:** 1 School of Medical Laboratory Sciences, Mizan-Tepi University, PO Box 260, Mizan-Aman, Ethiopia; 2 School of Medical Laboratory Sciences, Jimma University, Jimma, Ethiopia; 3 Departments of Epidemiology, Jimma University, Jimma, Ethiopia; 4 Schools of Veterinary Medicine, Jimma University, PO Box 378, Jimma, Ethiopia

**Keywords:** ESBLss, *E. coli*, Minced meat, Butcher shops, Jimma town

## Abstract

**Background:**

The impact of animals sources of food as a possible reservoir for extended-spectrum β-lactamase (ESBL) - Producing E. coli, and the dissemination of such strains into the food production chain need to be assessed. This study was aimed to assess the presence and antimicrobial susceptibility patterns of ESBLs - producing E. coli isolates from minced meat and environmental swab samples at meat retailer shops of Jimma town, Southwest Ethiopia.

**Methodology:**

A cross-sectional descriptive study was conducted from March to June, 2016. A total of 168 minced meat and swab samples were first enriched by buffered peptone water (BPW) for overnight and streaked onto MacConkey agar. Double disk synergy (DDS) method was used for detection of ESBL-producing strains. A disk of amoxicillin + clavulanic acid (20/10 μg) was placed in the center of Mueller-Hinton agar plate, and cefotaxime (30 μg) and ceftazidime (30 μg) were placed at a distance of 20 mm from the central disk. Checklist was used to assess hygienic status of butcher shops and practices meat handlers.

**Results:**

A total of 35 (20.80%) biochemically confirmed E. coli were obtained from 168 samples. Of these, 21 (23.9%) of them were detected from 88 minced meat and the other 14 (17.5%) from 80 swab samples taken from butcher’s hand, knives, chopping board and protective clothing. From 35 E. coli isolates, 7(20%) of them were confirmed as ESBL- producers. Among ESBL- producing strains, 85.7% were resistant for cefotaxime and ceftriaxone and 71.4% for ceftazidime. Among non-ESBLs-producing strains only seven isolates were resistant to third generation cephalosporin. All E. coli isolates were resistant to ampicillin, penicillin and erythromycin, and susceptible to amikacin. Poor hygienic status of butcher shops and unhygienic practice of meat handlers were observed.

**Conclusion:**

The detections of ESBLs- producing strains could be contributed for the increment of multi drug resistant isolates. This study also concluded that, unhygienic meat handling and processing practice can contribute for contaminations of meat. Thus, strategies should be planned and implemented to improve the knowledge and practice of butchers about handling and processing of meat.

## Background

Extended-Spectrum β- Lactamase (ESBLs)-producing *Escherichia coli* isolates have emerged as a global threat to human health, and have been isolated from human, animal and environment origins. The small but gradually increasing use of third generation cephalosporins in food animal production may be linked to the recent emergence of ESBLs-producing bacteria that associated with cattle, poultry and pigs [1, 2].

Extended-spectrum β-lactamase (ESBLs) is the current most important resistance mechanisms in *Enterobacteriaceae*. It can reduce the efficacy of modern expanded-spectrum cephalosporin and monobactams drugs, with the exceptions of cephamycins and carbapenems [3]. The emergence and spread of extended-spectrum β- lactamases (ESBLs) among members of *Enterobacteriaceae* family originating from food-producing animals and pets is a major public health issue worldwide. A conference held in 2010 by the Veterinary Laboratories Agency reported that ESBL-producing *Escherichia coli* now occurred on 37% of dairy farms sampled a ‘completely unexpected’ finding [4].

The use of antibiotics for growth promotion in livestock and for treatment of diseased animals may lead to the development of considerable resistant bacterial strains, in which it can be transmitted to humans through the food chain [5]. *Escherichia coli* has been considered a good indicator of the selective pressure exerted by the use of antimicrobials on the intestinal population of bacteria in food animals, and may also be used as a representative of the *Enterobacteriaceae* to monitor the emergence and changes in proportion of bacteria possessing extended-spectrum β- lactamase (ESBLs) [6–8].

A fresh red meats contains a high proportion of water and protein which provides a suitable environment for microbial growth [9]. The occurrence of *Escherichia coli* in foods of animal origin in Ethiopia is high due to many reasons like illegal slaughtering of animals in open fields, unhygienic slaughter practices, and the risk of disease due to this organism is high because of traditional practices of raw meat consumption. However, the precise attribution of animals and their food products as the sources of resistant strains, and the consequences of it on human health were not yet seriously evaluated. Thus, a study is designed to assess the presence of ESBL-producing *E. coli* from minced meat of cattle and from swab samples which is obtained from different meat retailer shops in Jimma town, Southwest Ethiopia.

## Methods

### Study design, area and period

Laboratory based cross- sectional descriptive study design was conducted in Jimma town, Southwest Ethiopia from March to June, 2016. Jimma town is found at 352Kms, Southwest of Addis Ababa, capital city of Ethiopia. The town is divided in to 17 administrative kebeles. According to the information obtained from Jimma town trade and industry office, the town has one municipality abattoir and around 120 meat retailer shops which directly receive slaughter service from the abattoir. Eighty eight (88) meat retailer shops were purposively selected from five most populated kebeles of the town (Mendera Kochi, Kosa Kito, Mentina, Hermata Merkato, and Bochobore) and the required samples were collected from those meat retailer shops operating during the study period in the five kebeles of the town.

### Laboratory sample collection

A total of 168 samples (88 minced meats of cattle and 80 swabs) were collected from meat retailer shops that operating during the study period in the five kebeles of the town. We made two visits of meat retailer shops per week for consecutive 4 weeks and within this period of time a total of 168 samples were collected. Eighty eight raw minced meat samples were collected from meat displayed for vending at the butcher shops. Twenty five grams (25 g) of minced meat samples were collected in sterile, separated plastic bags and labeled with permanent marker. The remaining 80 swab samples were also collected from hands of meat handlers, protective clothing, knives and chopping boards of the butcher shops at the beginning of operation. Swab samples were taken from 15 to 20 cm^2^ of the hands of meat handlers and protective clothing and from the surface of meat-cutting equipment such as knives and wooden chopping boards using sterile, buffered peptone water (BPW) moisten cotton swabs. The collected swab samples were returned into a separate test tube containing 9 ml sterile BPW, and all the collected samples were labeled, packaged in sterile plastic bags and carried to the microbiology laboratory of Jimma University in a cold box within 2 hrs of collection for processing [10].

**Minced meat:** is defined as a boneless meat that has been reduced to fragments [11].

### Sanitary status of butcher shops and hygienic practice of meat handlers

Sanitary status of the butcher shops and hygienic meat handling practices of workers were assessed by the use of observational checklist at Jimma town. Two visits of butcher shops were conducted per week for consecutive 4 weeks and the following variables were used: availability of clean cold and warm tap water, availability disinfectant and soap, regular hand washing practice during work, disinfection of the floor and processing tools before and during the work, protective clothing is clean, use of separate and washable chopping boards and knives for processing of abdominal organs and other parts of meat, whether the entire process was done in the same area without separation, whether the same buckets of water were used for cleaning knives, washing hands, whether butcher shops’ floor is constructed of concrete ceramic and has no crack, dust, rodent and insects and whether the meat displayed for vending protected from dust and flies.

### Isolation and identification process

A 25-g of minced meat of cattle were weighted and suspended into appropriate sterile cylinder/beakers that containing about 225 ml 0.1% buffered peptone water (BPW) and homogenized by shaking for 5 minutes in a sterile stomacher and incubated at 37 °C for 24 h for enrichment purposes. Similarly, all the collected swab samples were suspended into a test tube containing 9 ml sterile buffered peptone water (BPW) immediately at the collection sites and incubated at 37 °C for 24 h. After gently mixing, one loop-full (10 μl) of the overnight culture was streaked onto a MacConkey agar (Oxoid, UK) and incubated for 24 h at 37 °C [10].

### Identification of the isolates

The isolated colonies were first screened by their colony morphology, pink colour producing lactose fermenting colonies, gram staining techniques and further identified as *E. coli* by motility and other relevant biochemical tests, such as indole, citrate, urease, methyl red, gas and acid production tests. All the confirmed isolates were refrigerated at 2-8 °C until antimicrobial sensitivity test and ESBLs-production test was done.

### Screening of ESBL-producing isolates

The ESBL screening test was performed by standard disk diffusion method by using ceftazidime (30 μg) and cefotaxime (30 μg) disks. After adjusting 0.5 McFarland’s standard as indicated above, the suspension was inoculated onto Muller-Hinton agar (Oxoid, UK) with sterile cotton swab, and then the above two antibiotics disks were placed on the inoculated plate and incubated at 37 °C for 24 h. Isolates with reduced susceptibility to cefotaxime (≤ 27 mm) and ceftazidime (≤ 22 mm) around the disks were suspected as ESBLs*-* producers as recommended by CLSI guidelines [12].

### Conformation of ESBLs- producing isolates

Those isolates with reduced susceptibility to cefotaxime (≤ 27 mm) and ceftazidime (≤ 22 mm) were conformed for ESBLs*-* production using double disk approximation or double disk synergy (DDS) method. After inoculation of the suspension onto Muller-Hinton agar (MHA), a disk of amoxicillin + clavulanic acid (20/10 μg) was placed in center of the plate and then the disks of cefotaxime (30 μg) and ceftazidime (30 μg) were placed at a distance of 20 mm from the central disk on the same plate [12]. The plate was incubated at 37 °C for 24 h and examined for an enhancement of inhibition zone of the β-lactam drugs caused by the synergy of the clavulanate in the amoxicillin- clavulanate disk was interpreted as positive for ESBLs-production.

### Antibiotic sensitivity testing

The antibiotic sensitivity testing was performed by using Kirby-Bauer disc diffusion technique. The suspension of the growth were inoculated onto Muller-Hinton agar and cefotaxime (30 μg), Ceftriaxone (30 μg), ceftazidime (30 μg), amoxicillin-clavulanic acid (20/10 μg), penicillin (10 μg), ampicillin (10 μg), gentamycin (10 μg), amikacin **(**30 μg), neomycin (10 μg), ciprofloxacin (5 μg), erythromycin (15 μg), trimethoprim-sulfamethoxazole (1.25/23.75 μg), and tetracycline (30 μg) were placed on to the plate using sterile forceps. After overnight incubation of the plate at 37 °C, the zone of inhibition was measured by using sliding calipers and interpreted by comparing zone of inhibition with Kirby – Bauer chart as recommended by CLSI guidelines [12]. *K. pneumonia* ATCC* 700603 and *E. coli* ATCC*25922 were used as positive and negative control strains to monitor the quality of susceptibility testing and *ESBLs* detection methods. Multi drug resistance (MDR) is defined as a resistance to at least one agent in three or more antimicrobial classes [13].

### Data analysis

The data was analyzed by using SPSS version 16.0 with an emphasis on the human health significance of the findings. The difference in resistance between ESBL-producer and non-ESBL- producing strains were analyzed by using chi-square (*X*^2^) testing and *p-*value < 0.05 were regarded as statistically significant.

## Results

### Proportions of *E. coli* positive samples and ESBL- positive *E. coli* isolates

The current study, a total of 35 biochemically confirmed *E. coli* isolates were obtained from 20.8% (35/168) of specimens. Of these, 21 were isolated from 23.9% (21/88) of raw minced meat samples and the other 14 from 17.5% (14/80) of swab samples (i.e. 4 from 20% of hand swabs, 3 from 15% of knife swabs, 5 from 25% of chopping board swabs and 2 from 10% of protective clothing swabs). From the total 35 *E. coli* isolates 20% (7/35) of them were confirmed as ESBL- producers. The proportions of ESBL- producing strains against minced meat, hand swab and chopping board swab isolates were 23.8% (5/21), 25% (¼) and 20% (1/5), respectively (Table 1)*.*

**Table 1:** Proportions of *E. coli* positive samples and ESBLs- positive strains of *E. coli* in minced meat of cattle and swab samples obtained from different meat retailer shops in Jimma town, Southwest of Ethiopia

Types of samples	# of samples tested	Proportions of *E. coli* (+) samples (%)	Proportions of ESBLs-positives *E. coli* isolates (%)	Proportions of ESBLs-negative *E. coli* isolates (%)
Minced meat	88	21 (23.9%)	5 (23.8%)	16 (76.2%)
Hand swabs	20	4 (20%)	1 (25%)	3 (75%)
Knives swabs	20	3 (15%)	0	3 (100%)
Chopping board swabs	20	5 (25%)	1 (20%)	4 (80%)
Protective clothing swabs	20	2 (10%)	0	2 (100%)
Total	168	35 (20.8%)	7 (20%)	28 (80%)

### Sanitary status of butcher shops and hygienic practice of meat handlers

In our observational checklist survey, only 32 (36.4%) of the floors were made of concrete ceramic and only 29 (33%) of the floors were free of cracks (Table 2). Although, 72 (81.8%) of the butcher shops have ceiling (local name; ), only 27 (30.7%) of them were properly finished and free of dusts. Only 7 (8%) butcher shops have insect and dust proof shelf for meat display and only 17 (19.3%) of them were having smooth and easily washable chopping board for cutting of meats. Only 30 (34.1%) of butcher shops use clean knives and clean meat hanger.

**Table 2:** Summary of observational checklist survey on sanitary status of the meat retailer shops and hygienic practice of meat handlers in Jimma town, Southwest Ethiopia

Variables (checklists)	# of butcher shops checked = 88	
Meat handlers practices	Frequency	%
Use of protective clean clothing	9	10.2%
Meat handling training	0	0
Handling money with bare hands	73	83%
Wash hand basin nearby	2	2.3%
Use of plastic bag, newspaper to wrap meat	88	100%
Regular washing of hand & processing tools	2	2.3%
Regular disinfection of hand, the floor and processing tools	0	0
Use of separate chopping boards (flat tables) for cutting of meat and internal and/or abdominal organs	22	25%
Use of separate knives for cutting of meat and abdominal contents	22	25%
Use of the same buckets of water for cleaning knives, washing hands	61	69.3%
Hygienic status of the butcher shops premises, utensils		
Floor is made of concrete ceramic	32	36.4%
Floor free of cracks	29	33%
Having ceiling (local name; )	72	81.8%
Wall and ceiling free of dust	27	30.7%
Wall painted white color paint	76	86.4%
Having insect and dust proof shelf for meat display	7	8%
Having washable chopping board	17	19.3%
Clean knives	30	34.1%
Clean meat hanger	31	35.2%

In our observations, from the 88 meat retailer shop’s workers, only 9 (10.2%) of them wore clean protective clothing during meat handling. None of the butchers had taken any form of formal training on safe handling of meat and hygienic practices. About 73 (83%) the butchers were handling money with bare hands during their works and only 2 (2.3%) were have wash hand basin nearby and regular hand washing, processing tools practices. However, none of the butchers have disinfection practice of hands, the floor and processing tools and only 22 (25%) of them were used separate chopping boards (flat wooden tables) and knives for cutting of meat and internal and/or abdominal organs (Table 2).

### Antibiotic resistance profile

In our study, all 35 isolates of *E. coli* tested for their antibiotic resistance profile against 13 different antimicrobial agents (Table 3). Significant differences in resistance to third generation cephalosporin were observed among ESBL*-*producers and non- ESBL*-*producers (*p* < 0.05). Among ESBL*-*producers 6 (85.7%) of the isolates were resistant to cefotaxime and ceftriaxone and 5 (71.4%) of them were resistant to ceftazidime. Among non-ESBLs-producing strains only seven isolates were resistant to third generation cephalosporin; 6 were resistant to ceftazidime and only one was resistant to cefotaxime. All *E. coli* isolates were resistant to ampicillin, penicillin and erythromycin, and all of them were susceptible to amikacin (Table 3).

**Table 3:** Antibiotics resistance profiles of *E. coli* isolates in minced meat and swab samples obtained from different meat retailer shops of Jimma town, Southwest of Ethiopia

Antibiotics	Total R (%)	ESBL- positive (*n* = 7)		ESBL-negative (*n* = 28)		*p -*value
		R (%)	S (%)	R (%)	S (%)	
Cefotaxime	7 (20.0)	6 (85.7)	1 (14.3)	1 (3.6)	27 (96.4)	0.001
Ceftazidime	11 (31.4)	5 (71.4)	2 (28.6)	6 (21.4)	22 (78.6)	0.021
Ceftriaxone	6 (17.1)	6 (85.7)	1 (14.3)	0	28 (100)	0.001
AMC	11 (31.4)	6 (85.7)	1 (14.3)	5 (17.9)	23 (82.1)	0.002
Ampicillin	35 (100)	7 (100)	0	28 (100)	0	–
Penicillin	35 (100)	7 (100)	0	28 (100)	0	–
Gentamycin	3 (8.6)	1 (14.3)	6 (85.7)	2 (7.1)	26 (92.9)	0.499
Amikacin	0	0	7 (100)	0	28 (100)	–
Ciprofloxacin	4 (11.4)	1 (14.3)	6 (85.7)	3 (10.7)	25 (89.3)	1.000
Tetracycline	19 (54.3)	6 (85.7)	1 (14.3)	13 (46.4)	15 (53.6)	0.096
SXT	19 (54.3)	6 (85.7)	1 (14.3)	13 (46.4)	15 (53.6)	0.096
Erythromycin	35 (100)	7 (100)	0	28 (100)	0	–
Neomycin	1	1 (14.3)	6 (85.7)	0	28 (100)	0.200

*R*: Resistant, *S*: Sensitive, *AMC*: Amoxicillin-clavulanic acid, *SXT*: Trimethoprim- sulphamethoxazole

### Multi- drug resistance (MDR) profiles

In this study, 26 (74.3%) of *E. coli* isolates were resistant to three or more classes of antibiotics, such as tetracycline, cotrimoxazole and erythromycin plus β- lactam group of antibiotics. The most frequent resistant phenotype was for ampicillin, penicillin and erythromycin, followed by the above antibiotics plus cotrimoxazole and tetracycline. The resistance against three, four, five, six and seven antibiotic classes were 9 (34.6%), 8 (30.8%), 5(19.2%), 3(11.5%) and 1(3.5%), respectively (Table 4).

**Table 4:** Multi-drug resistance profiles of *E. coli* isolated from minced meat and swab samples obtained from different meat retailer shops of Jimma town, Southwest of Ethiopia

Resistant patterns	# of antibiotics classes	Total R (%)
Amp, P, ERY, T	3	9 (34.6%)
Amp, P, ERY, SXT		
Amp, P, ERY, CS, AMC, T	4	8 (30.8%)
Amp, P, ERY, SXT, T		
Amp, P, ERY, CIP, SXT		
Amp, P, ERY, CS, SXT, T	5	5(19.2%)
Amp, P, ERY, CAZ, SXT, T		
Amp, P, ERY, CS, AMC, SXT, T		
Amp, P, ERY, CAZ, AMC, SXT, T		
Amp, P, ERY, CS, AMC, GEN, N, SXT, T	6	3 (11.5%)
Amp, P, ERY, CAZ, CIP, SXT, T		
Amp, P, ERY, CS, AMC, GEN, SXT, T		
Amp, P, ERY, CS, AMC, CIP, SXT, T		
Amp, P, ERY, CAZ, AMC, GEN, CIP, SXT, T	7	1(3.8%)

***Amp*** ampicillin, ***P*** penicillin, ***ERY*** erythromycin, ***CS*** (cefotaxime, ceftazidime and ceftriaxone), ***GEN*** gentamycin, ***N*** neomycin, ***CIP*** ciprofloxacin, ***CAZ*** ceftazidime, ***SXT*** trimethoprim-sulphamethoxazole, ***AMC*** amoxicillin-clavulanic acid, ***T*** tetracycline

## Discussion

Meat passed through different contacts starting from slaughter house until it consumed as food, which increase its chance of antimicrobial contamination. However, little information is available concerning the prevalence of the resistant strains of *E. coli* in raw meat and environmental samples from meat retailer shops in the study area as well as in the country, Ethiopia. The current study revealed that, a total of 35 biochemically confirmed *E. coli* isolates were obtained from 21(23.9%) of minced meat samples and 14 (17.5%) of swab samples. Of these, 7 (20%) of them were confirmed as ESBLs- producers. This is the first report presenting data on meat sources of ESBLs*-* producing *E. coli* in our setting. Thus, comparison of our results from different countries is may not be feasible due to differences in the study methodology, such as method of enrichment and isolation procedures, differences in sample size and type of sample and how and when it was collected. However, the proportion of ESBL- producing *E. coli* strains obtained in this study was lower than the study finding in Vietnam [14] and Pakistan [15] and higher than the study finding in China [16]. In addition to the above methodological differences, the observed differences might be also related with the frequent and miss-use practices of third generation cephalosporins in humans and food animal, since the use of these antibiotics greatly linked to the recent emergence of ESBLs-producing bacteria.

The total *E. coli* isolates obtained in this study is higher than the study finding in Sothern [17] and Eastern [18, 19], Ethiopia and slightly lower than the study finding in different parts of Ethiopia [20–22]. The difference observed in the prevalence of *E. coli* could be due to the differences in the sanitary standards of meat retailer shops premises and hygienic practices of meat handlers. The low sanitary standard of meat retailer shops and poor hygienic practices of meat handlers observed in our survey might be contributing to cross-contamination of meat with *E. coli* isolates.

The occurrence of *E. coli* in the raw minced meat samples in this study was (23.9%), which was the highest proportion as compared with hand, knife, chopping board and protective clothing swab samples. This is the fact that, fresh red meats contains a high proportion of internal water and protein activities, which provides a suitable environment for microbial growth [9]. Surface contamination on the carcass can be transferred to meat cuts via meat processing equipment as the animal is further processed [9]. When primal cuts of meats are further processed (e.g. minced/ground meat, sausages), microorganisms are homogenized throughout the product. This is why higher proportions minced meat samples were positive for *E. coli* in this study. A study from Turkey also showed that, almost all of minced meat samples analyzed were contaminated with *E. coli* [23]. However, there are no regular inspections of meat retailer shops and abattoirs in our setting to protect meat from contaminations. This is might be posing great problems especially because of the wide spread practice of eating raw meat throughout the country.

Intestinal carriage of *E. coli,* including ESBLs- producing strains in food-producing animals may leads to contamination of retail meat [24]. A study in France indicates a worrisome prevalence of fecal carriage of cephalosporin resistance in cattle, with a higher prevalence of *ESBL*-producing *E*. *coli* at slaughterhouses compared to farms [25]. Improper handling, processing and display of meat at the slaughtering places and at butcher shops can affect the quality of meat which indicated as the presence of cross-contamination [26]. Thus, the presence of resistant strain of *E. coli* in meat of cattle and swab samples might be due to cross-contaminations either from caecal contents of animals and/or from different contaminated materials and hands of meat handlers.

The summary of our observational checklist survey showed that, most of butcher shops were didn’t fulfill the requirements of WHO and FAO food safety standards [27]. For example, more than half of butcher shops’ floors and walls were not constructed from materials that are easily washable and only 33% of the floors were free of cracks. Although, more than 80% of the butcher shops have ceiling (local name; ), only 30.7% of them were free of dusts and insects. In additions, only 8% butcher shops have insect and dust proof shelf for meat display and the more than 80% of meat retailer shops’ chopping board was made of wooden material with no easily washable surface. Poor hygienic status of butcher shops and unhygienic practice of meat handlers were also reported in other study in Gondar and Somali region of Ethiopia [28, 29].

None of the butchers had taken any form of formal training on meat safety and hygiene. In addition, none of them have had regular disinfection practices of hands, the floor and processing tools and only one fourth of them were use of separate chopping boards (flat wooden tables) and knives for cutting of internal and/or abdominal organs that have contaminated with caecal contents and other parts of the meats. Eighty three percent (83%) of the butchers were handled money with their bare hands during selling of meat. It is well documented facts that unhygienic practice is one of the most important sources of contamination for foods [13, 26, 30]. For instance, simultaneous handling of food and money increases the risk of cross contamination of meat [31]. Thus, the range of activities involved in meat hygiene should be carried out by responsible bodies with the appropriate training on knowledge and hygienic practices of meat handlers.

In the current study, higher resistance rate were observed in ESBLs-producers toward third generation cephalosporin *(p –*value < 0.05). However, there is no difference in other classes of antibiotics agents tested. In this study 26 (74.3%) of *E. coli* isolates were showed co-resistance to three or more classes of antibiotics, such as tetracycline, co-trimoxazole and erythromycin plus β- lactam group of antibiotics. The higher rate of multi-drug resistant (MDR) isolates were also reported in other area of Ethiopia [32], Vietnam [14] and Pakistan [15], in which (56.5 to 96.3%) of ESBLs-producing and non- ESBLs-producing *E. coli* isolates were showed multiple drug resistance for three or more antibiotics. The result of this study, combined with data from previous studies in different countries, suggests that unhygienic practice of food handling and processing is major contributors to the dissemination of not only ESBL- *E. coli* but also MDR bacteria. Thus, the development of stringent monitoring strategies and the promotion of hygienic meat distribution practices are needed to control the spread of these antibiotic-resistant bacteria.

In the present study, lower resistance was observed against gentamycin, neomycin and ciprofloxacin and no resistance was seen with amikacin. In contrast, all *E. coli* isolates of meat and swab samples were resistant to ampicillin, penicillin and erythromycin and more than 50% of the isolates were resistant to tetracycline and co-trimoxazole. This finding is comparable with the previous reports in Eastern part of Ethiopia, in which the prevalence of resistance among *E. coli* isolates of meat to penicillin, ampicillin, erythromycin and tetracycline were ranging from (33.3 to 100%) [19, 22]. This situation has dragged our condition towards increased load of antibiotics, poor clinical outcome and limited therapeutic options.

## Conclusion

In the present study, the presence of ESBLs-producing strains may contributed for the occurrence of multi-drug resistant isolates to many classes of antibiotics, such as ampicillin, penicillin and erythromycin, SXT and tetracycline. In addition, the low sanitary standard of butcher shops and unhygienic practices of meat handlers observed may have implications for contaminations of meat with *E. coli* isolates. Therefore, strategies should be planned and implemented to improve the knowledge and practice of butchers about handling and processing of meat. Moreover, monitoring the prevalence of antimicrobial resistance among isolates from healthy animals and their food products provide evident data for designing strategy on prevention and control of this resistant strain from spread in the community.

## Data Availability

“All the data supporting our findings were incorporated within the manuscript.”
